# Tissue specific CD4^+^ T cell priming determines the requirement for interleukin-23 in experimental arthritis

**DOI:** 10.1186/s13075-014-0440-1

**Published:** 2014-09-25

**Authors:** Susan A Olalekan, Yanxia Cao, Alison Finnegan

**Affiliations:** Department of Immunology/Microbiology, Rush University Medical Center, Cohn Research Building, Chicago, IL 60612 USA; Department of Medicine, Division of Rheumatology, Rush University Medical Center, 1735 West Harrison Avenue, Chicago, IL 60612 USA

## Abstract

**Introduction:**

Rheumatoid arthritis (RA) is a chronic inflammatory disease with striking heterogeneity in (i) clinical presentation, (ii) autoantibody profiles and (iii) responses to treatment suggesting that distinct molecular mechanisms may underlie the disease process. Proteoglycan-induced arthritis (PGIA) is induced by two pathways either by intraperitoneal (i.p.) or subcutaneous (s.c.) exposure to PG. CD4^+^ T cells primed by the i.p. route are T helper (Th)1 cells expressing interferon gamma (IFN-γ) whereas CD4^+^ T cells primed by the s.c. route are Th17 cells expressing interleukin (IL)-17. IL-23 is necessary for maintaining the phenotype of Th17 cells; however, IL-23 is inflammatory independent of IL-17. The aim of this study was to determine if PGIA induced by different routes of immunization is dependent on IL-23.

**Methods:**

BALB/c wild type (WT), IL-12p40^−/−^ and IL-23p19^−/−^ littermate mice were immunized with recombinant G1 (rG1) domain of human PG in adjuvant either i.p. or s.c. and development of arthritis monitored. Joint histology was assessed. CD4^+^ T cell cytokines in spleen, lymph node (LN), and joint were assessed by intracellular staining and cytokine enzyme-linked immunosorbent assay. RNA transcripts for cytokines and transcription factors were examined.

**Results:**

PGIA was suppressed in the p40^−/−^ and p19^−/−^ mice immunized by the s.c. route but only inhibited in p40^−/−^ mice by the i.p. route. The joints of s.c. but not i.p. sensitized mice contained a population of CD4^+^ T cells expressing single positive IFN-γ and IL-17 and double positive IFN-γ/IL-17 which were dependent on IL-23 expression. The IFN-γ and IL-17 response in spleen and inguinal LN was inhibited in p19^−/−^ mice and p40^−/−^ mice after s.c. immunization, whereas in i.p. immunized p19^−/−^ mice, IL-17 but not IFN-γ was reduced. Inguinal LN CD11c^+^ dendritic cells (DC) from s.c. immunized, but not spleen DC from i.p. immunized mice, produced IL-23, IL-1β, and IL-6 and activated T cells to produce IL-17.

**Conclusion:**

IL-23 is necessary for the activity of Th17 after s.c. immunization and does not play a role independent of IL-17 after i.p. immunization. These data demonstrate that the molecular pathways IL-23/17 and IL-12/IFN-γ may represent subtypes of arthritis determined by the mode of induction.

**Electronic supplementary material:**

The online version of this article (doi:10.1186/s13075-014-0440-1) contains supplementary material, which is available to authorized users.

## Introduction

Rheumatoid arthritis (RA) is a chronic inflammatory disease affecting synovial tissue in multiple joints characterized by infiltration of leukocytes into the synovial lining and hyperplasia of the resident synoviocytes. The clinical presentation of RA reveals striking heterogeneity; moreover, patients with apparently identical clinical involvement may have very dissimilar patterns of leukocyte infiltration and activation of genes associated with inflammation [1,2]. This heterogeneity extends to therapy, where even with the advent of highly effective biologically based therapeutics such as tumor necrosis factor blockade [3,4], anti-CD20 monoclonal antibodies [5], CTLA-4 co-stimulation inhibition [6], and interleukin (IL)-6 inhibition [7] at best 40 to 50% of subjects achieve an American College of Rheumatology 50% improvement criteria response with any specific agent [8]. These data support the concept that RA may be initiated by different pathogenic processes, each of which leads to a common final pathway – joint damage.

How RA is initiated is unclear, but T-cell responses to self-antigens are implicated based on the strong linkage of RA to particular MHC alleles. Cytokines produced by CD4^+^ T cells play a central role in orchestrating immune responses. CD4^+^ T cells involved in inflammatory responses are divided into T-helper (Th) 1 cells that produce interferon gamma (IFNγ) and Th17 cells that produce IL-17A, IL-17F and IL-22 [9,10]. The differentiation of Th0 cells is initiated by innate immune cells activated to release proinflammatory cytokines; IL-12 and IFNγ promote Th1 cells, whereas transforming growth factor beta (TGFβ), IL-6, and IL-1β promote Th17 differentiation [11-14]. IL-23 is dispensable for Th17 differentiation but is required to enhance and maintain the Th17 phenotype [15,16]. IL-12 and IL-23 are heterodimeric cytokines that share a common p40 subunit which pairs with p35 (IL-12) and with p19 (IL-23) [17,18]. T-cell-mediated autoimmune diseases were originally perceived to be driven by Th1 IFNγ production based on the evidence that p40-deficient mice and antibodies specific for p40 inhibited experimental autoimmune encephalomyelitis (EAE), collagen-induced arthritis (CIA), and experimental autoimmune uveitis (EAU) [19-22]. However, paradoxically mice lacking components of the Th1 pathway – IFNγ, IFNγ receptor, and IL-12p35 – experienced exacerbated EAE, CIA, and EAU [23-26]. This discrepancy was resolved by the identification of p19, the second binding partner for p40. Studies revealed that mice deficient in IL-23p19 have reduced IL-17 expression, establishing a link between IL-23 and IL-17 [16,18]. Mice genetically deficient in IL-23p19 are resistant to EAE and CIA [25,26]. Genetic deficiency in IL-17 and IL-17 neutralization studies demonstrates a role for IL-17 in EAE, CIA, and EAU [25-29]. These studies led to the concept that the pathogenic effects previously attributed to the IL-12/IFNγ pathway are mediated by IL-23 and IL-23-driven Th17 effector cells. However, it is now known that other immune cells can respond to IL-23 [30]. IL-23 can also mediate osteoclastogenesis independent of IL-17 [31]. In addition, systemic induction of IL-23 induces entheseal inflammation in a model of ankylosing spondylitis that is independent of IL-17 [32].

Contrary to the requirements for IL-17 in EAE, CIA, and EAU, proteoglycan-induced arthritis (PGIA) was originally described as a Th1-mediated disease. Genetic deficiency in IL-12, IFNγ, the IL-27 receptor and Stat4 reduced susceptibility to arthritis whereas a deficiency in IL-17 had no effect on disease [33-37]. We recently identified the basis for the difference between these autoimmune models. We found that altering the route of immunization from intraperitoneal (i.p.) to intradermal/subcutaneous (i.d./s.c.; designated s.c. hereafter) alters the CD4^+^ T-cell differentiation pathway from Th1 to Th17 [38]. CD4^+^ T cells primed by the i.p. route differentiate into IFNγ-expressing T cells with very few IL-17-producing cells, whereas CD4^+^ T cells primed by the s.c. route produce both IFNγ and IL-17. Importantly, the histological picture of arthritis induced by either the i.p. route (Th1) or the s.c. route (Th17) appears to be similar. These data suggest that different pathways of arthritis induction can lead to similar manifestation of joint destruction. CIA, EAE, and EAU in general are induced by s.c. immunization, suggesting that the route of immunization determines the Th17 pathology of these autoimmune models. Since IL-23 is an important cytokine driving several autoimmune diseases, this raises the question of whether IL-23 can drive inflammation in a model of arthritis that is independent of IL-17 as it occurs in ankylosing spondylitis [30]. We report in this study that the microenvironment in which CD4^+^ T cells were initially primed to proteoglycan (PG) dictates the requirement for IL-23. We found that IL-23 was required for the development of arthritis induced by s.c. immunization but not the i.p. route. Remarkably, IL-23 appeared to be necessary for both T-cell IL-17 and IFNγ production in s.c. immunized mice. These data demonstrate that the ability to generate the appropriate signal in a specific contextual environment of naïve CD4^+^ T-cell differentiation is different at distinct anatomical sites. Furthermore, these data suggest that the molecular pathways IL-23/17 and IL 12/IFNγ may represent particular subtypes of arthritis determined by the tissue site of self-antigen exposure.

## Methods

### Animals

C57BL/6 IL-23p19-deficient mice were obtained from Genentech (San Francisco, CA, USA) and backcrossed to BALB/c mice obtained from National Cancer Institute (Bethesda, MD, USA) for eight generations, and were then intercrossed to obtain wild type (WT) and p19^−/−^ littermates. WT and p19^−/−^ littermates were used in all experiments. WT BALB/c and BALB/c IL-12p40-deficient mice were obtained from Jackson Laboratories (Bar Harbor, ME, USA). T-cell receptor transgenic TCR-Tg 5/4E8 mice, specific for an immunodominant peptide in the human G1 domain of PG that cross-reacts with mouse G1, were generated as described previously [39]. Female mice <3 months were used in all experiments. Animal experiments were approved by the Institutional Animal Care and Use Committee and the Institutional Biosafety Committee.

### Induction and assessment of arthritis

Recombinant human aggregan G1-domain (rG1) protein consisting of 351 amino acids of the G1 domain and 59 amino acids of the interglobular domain of PG was cloned from human chondrocytes. A fusion protein consisting of human G1 domain cDNA and the mouse IgG_2a_-Fc fragment was inserted into the mammalian expression vector Lonza pEE14.1 plasmid. Protein G-sepharose was used for G1/Fc purification. The quantity and purity of the rG1 domain was determined on western blot by anti-G1 antibody (G18) [40].

PGIA was induced by immunization of mice via either the i.p. or s.c. route with 40 μg rG1 protein in 1 mg dimethyldioctadecyl ammonium bromide (Sigma-Aldrich, St. Louis, MO, USA) in 100 μl. Mice were boosted at 3 and 6 weeks. Induction of arthritis was monitored over time by biweekly scoring in a blinded manner. The paws were scored using an established scoring system on a scale of one to four: 0, normal; 1, mild erythema and swelling of several digits or part of the paw; 2, moderate erythema and swelling of digits and paw; 3, more diffuse erythema and swelling; and 4, severe erythema and swelling of the complete paw with ankylosis. The incidence of arthritis indicates the percentage of mice that develop arthritis. Individual paws score 0 to 4, with a cumulative score ranging from 0 to 16. Groups of mice (*n* =11 to 12) were immunized and the experiments were repeated. The arthritis score represents the mean ± standard error of the mean of the data. Histology was performed on hind ankle joints of immunized mice. Joints were fixed in 10% neutralizing formalin, decalcified in 5% formic acid, and embedded in paraffin. At least three sections per paw (~200 μm apart) were stained with haemotoxylin and eosin. Cellular infiltration and bone erosion were measured on a scale of 0 to 4 by a blinded observer, and values represent means ± standard deviation.

### Flow cytometry analysis

Spleen and inguinal lymph node (LN) cells were harvested from immunized mice at the time of sacrifice. Joint tissues were minced and treated with collagenase (0.25 mg/ml), and joint cell populations were examined for surface markers using antibodies anti-CD4-AF700, anti-CD8a-PE-Cy7, anti-CD19-PE, anti-CD11b-Pacific Blue, anti-CD11c-APC-Cy7, anti-F4/80-APC, and anti-GR-1-FITC (BD Pharmingen, San Jose, CA, USA). For intracellular cytokine staining, cells were stimulated with Phorbol 12-myristate 13-acetate (25 ng/ml) and ionomycin (500 ng/ml) (Sigma-Aldrich) and treated with GolgiPlug (BD Pharmingen) for 4 hours. After cell surface staining with anti-CD3e-PE-Cy7 and anti-CD4-APC-Cy7, cells were permeabilized using the Cytofix/Cytoperm Plus kit (BD Pharmingen) and stained with anti-IFNγ-APC and anti-IL-17A-FITC. A BD LSRII cytometer was used for cytometry and data were analyzed using BD FACS Diva Software.

### Cytokine production and T-cell proliferation

Spleen and inguinal LN were harvested from immunized mice either from arthritic mice approximately 70 days after the first immunization or from mice 9 days after immunization. Spleen and inguinal LN cells (5.0 × 10^5^/well) were incubated in the presence and absence of rG1 (2 μg/ml). CD4^+^ T cells (2.5 × 10^5^), prepared by negative selection (Miltenyi Biotec, Auburn, CA, USA), were incubated with mitomycin-treated naïve spleen cells (2.5 × 10^5^) as antigen-presenting cells in the presence and absence of rG1 (2 μg/ml). Cells were cultured in RPMI 1640 medium containing 5% fetal bovine serum and supplemented with 100 μg/ml streptomycin, 100 U/ml penicillin, and 2 mM l-glutamine for 4 days and supernatants were harvested for cytokine detection by enzyme-linked immunosorbent assay (R&D Systems, Minneapolis, MN, USA). Proliferation was measured by the incorporation of ^3^H-thymidine for the final 24 hours.

### CD11c dendritic cell activation

Mice were immunized with rG1/dimethyldioctadecyl ammonium bromide by either the i.p. or s.c. route. Spleen and inguinal LN tissues were harvested 24 hours later and treated with collagenase (0.25 mg/ml) to release tissue adherent dendritic cells (DCs). CD11c DCs were positively selected using microbeads (Miltenyi Biotec) and then sorted using the MoFlo Legacy cell sorter (Beckman Coulter, Brea, CA USA). CD4^+^ T cells were enriched from single cell suspensions of spleen cells from TCR-Tg (5/4E8) mice by negative selection using microbeads (Miltenyi Biotec).

### Detection of anti-G1 specific antibodies

Serum was obtained from immunized mice at approximately 70 days after the first immunization by bleeding mice from the orbital plexus. The presence of anti-G1 antibodies in serum samples was determined by enzyme-linked immunosorbent assay in serial diluted (PBS/0.5% Tween-20) samples and internal standard samples (pooled sera from arthritis mice) as described [41].

### Quantitative reverse transcription-polymerase chain reaction

Spleen and inguinal LN were harvested 6 hours and 12 hours after i.p. and s.c. immunization for the isolation of sorted CD11c^+^ DCs or at 5 days for the isolation of CD4^+^ T cells. RNA was isolated using TRIzol Reagent (Life Technologies, Carlsbad, CA, USA). Reverse transcription was performed with qScript cDNA SuperMix (Quanta Biosciences, Gaithersburg, MD, USA). Gene-specific amplification was performed using Perfecta SYBR Green SuperMix (Quanta Biosciences) and normalized to β-actin levels for each sample. All samples were run in triplicate on a BioRad CFX96 machine using BioRad proprietary software (Bio-Rad, Hercules, CA USA). To confirm that the same amount of RNA was added to each polymerase chain reaction, murine β-actin amplification was performed on each sample. Relative fold induction was calculated using the formula 2^–(ΔΔ*C*t)^, where ΔΔ*C*T is Δ*C*T_(treatment)_ – Δ*C*T_(control)_, Δ*C*T is *C*T_(target gene)_ – *C*T_(β actin)_, and *C*T is the cycle at which the threshold is crossed. Polymerase chain reaction product quality was monitored using post-polymerase chain reaction melt curve analysis. Controls were from naïve nonimmunized spleen and inguinal LN. Fold increases were normalized to naïve cell populations.

### Statistical analysis

All significance was determined using computer-based statistics software (SPSS, Chicago, IL, USA). The Mann–Whitney U test was used to compare nonparametric data for statistical significance, with two-tailed *P* values indicated throughout as *P* <0.05.

## Results

### IL-23 is critical for the development of PGIA after subcutaneous but not intraperitoneal induction

**Figure 1:**
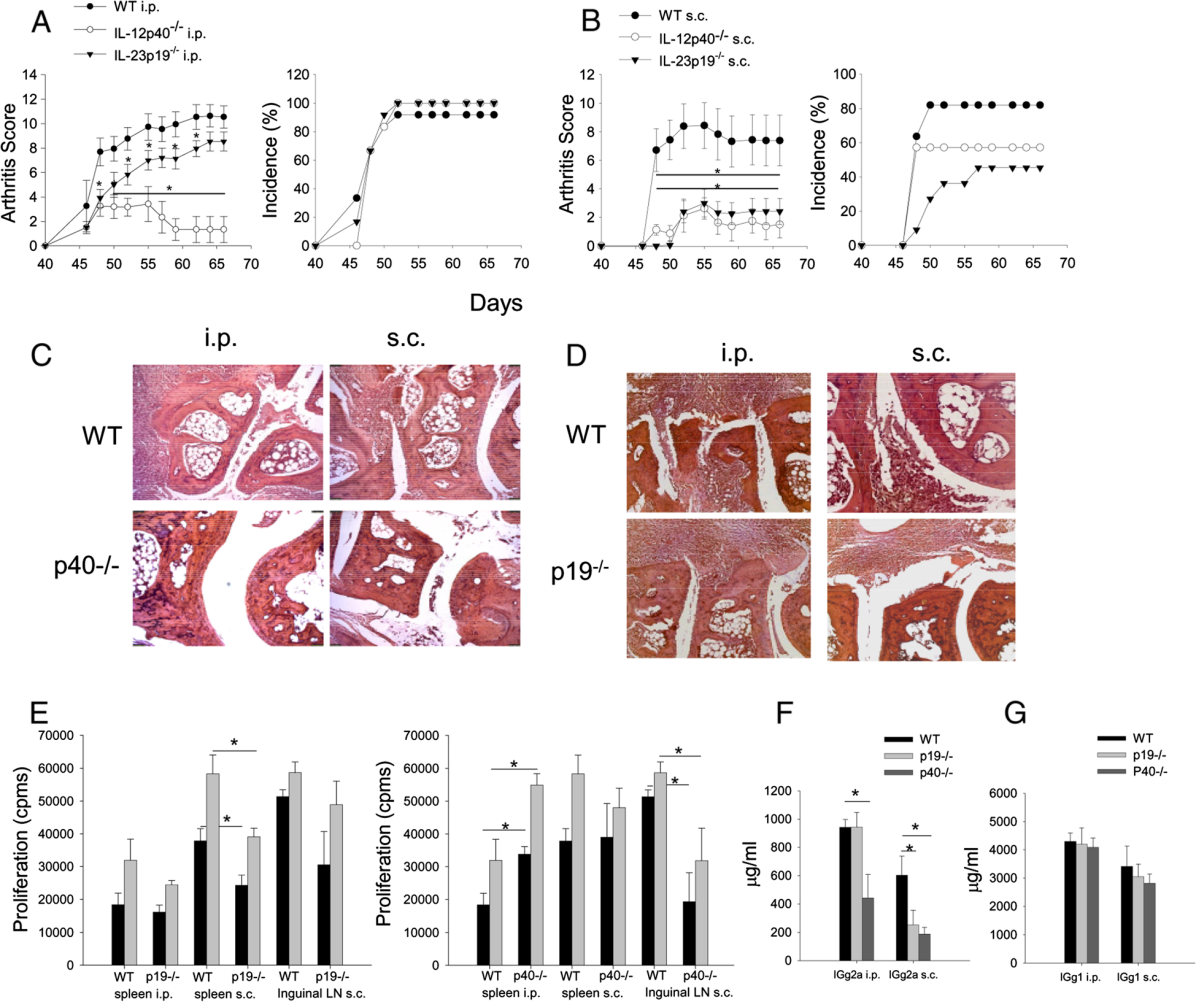
**Arthritis and joint inflammation is suppressed in subcutaneous immunized IL-23p19** ^**−/−**^ **mice.** Arthritis severity and incidence over time after immunization of wild type (WT) BALB/c, p40^−/−^ and p19^−/−^ mice either by **(A)** intraperitoneal (i.p.) or **(B)** intradermal/subcutaneous (i.d./s.c.) route three times with rG1 in dimethyldioctadecyl ammonium bromide. Data represent mean ± standard error of the mean (SEM, *n* =11 to 12 mice) and are representative of three independent experiments. *P <0.05. Representative images of ankle joints of **(C)** WT and IL-12p40^−/−^ i.p. immunized mice and **(D)** WT and IL-23p19^−/−^ s.c. immunized mice. Ankle joints were dissected, fixed in formalin, decalcified, embedded in paraffin and stained with haemotoxylin and eosin. **(E)** Proliferation of spleen and inguinal lymph node (LN) from WT, p40^−/−^ and p19^−/−^ mice. Spleen and inguinal LN were harvested from immunized mice at approximately 70 days. Cells were restimulated *in vitro* with rG1 for 4 days and proliferation measured as the incorporation of ^3^H-thymidine (*n* =11 to 12). **(F)** G1-specific IgG_2a_ antibody and **(G)** G1-specific IgG_1_ were measured in sera by enzyme-linked immunosorbent assay (*n* =4 to 5). Data represent mean ± SEM. *P <0.05. IL, interleukin.

We have reported that PGIA is induced by immunization with PG in adjuvant by either the i.p. or the s.c. route [38]. Induction by the i.p. route is dependent on IFNγ whereas induction by the s.c. route is IL-17 dependent. In addition, IL-12 (p40^−/−^) mice are resistant to induction of arthritis sensitized by the i.p. route [37]. Since p40 is a shared subunit of IL-12 and IL-23, and if IL-23 is involved in the Th17 pathway induced by the s.c. route, p40^−/−^ mice should be resistant to the induction of arthritis by the s.c. route. WT and p40^−/−^ mice were immunized by the i.p. or s.c. route with rG1 in adjuvant and development of arthritis was monitored over time. Arthritis was significantly suppressed in p40^−/−^ mice immunized either by i.p. or s.c. route (Figure 1A,B). Histology of the ankle joints of WT and p40^−/−^ mice demonstrated reduced infiltration of inflammatory cells and joint damage (Figure 1C). Resistance to arthritis after s.c. immunization in p40^−/−^ mice suggests a requirement for IL-23 in the induction of PGIA by the s.c. route. To determine the requirement of IL-23 in the induction of PGIA by the s.c. route, BALB/c WT and p19^−/−^ littermate mice were immunized either by the i.p. or s.c. route with rG1 in adjuvant. WT mice immunized by the i.p. or s.c. route developed arthritis in a similar manner, with the s.c. route slightly less robust than the i.p route (Figure 1A,B). The i.p. immunized p19^−/−^ mice developed inflammation although it was reduced in comparison with WT mice (Figure 1A). In the p19^−/−^ mice immunized by the s.c. route, arthritis was significantly inhibited with only 40% of the mice developing very mild arthritis (Figure 1B). To determine whether the appearance of erythema and swelling corresponded to cellular infiltration and joint damage, we examined joint histology from hind ankle joints (Figure 1D). In WT mice immunized by either the i.p. or s.c. route there was a similar degree of cellular infiltration and bone erosion that scored a maximum of 4 for all ankle joints. However, the reduction of inflammation in the p19^−/−^ after s.c. immunization corresponded to a significant reduction in cellular infiltration and bone erosion with a score of 1.3 ± 1.5 (*P* <0.05). There was very little clinical difference in the WT mice immunized by either the i.p. or s.c. route. The degree of severity and the incidence of disease were somewhat more robust in the i.p. versus s.c. route of immunization, but this can vary between different experiments. The onset of arthritis and the pattern of joint involvement were no different between i.p. and s.c. sensitization.

We next assessed whether the reduction in arthritis was due to reduced T-cell activation and PG-specific antibody production. Although there were some significant differences in T-cell proliferation between WT and p40^−/−^ or p19^−/−^ i.p. and s.c. immunized mice, there was no correlation with resistance to PGIA (Figure 1E). However, the G1-specific IgG_2a_ antibody response correlated with the reduction in arthritis. There was a reduction in the IgG_2a_ anti-G1 antibody response in s.c. sensitized p19^−/−^ and p40^−/−^ mice in comparison with WT mice but only in p40^−/−^ mice after i.p. sensitization (Figure 1F,G).

### An IFNγ/IL-17 double-positive CD4^+^ T-cell population in the joint tissues is regulated by IL-23

**Figure 2:**
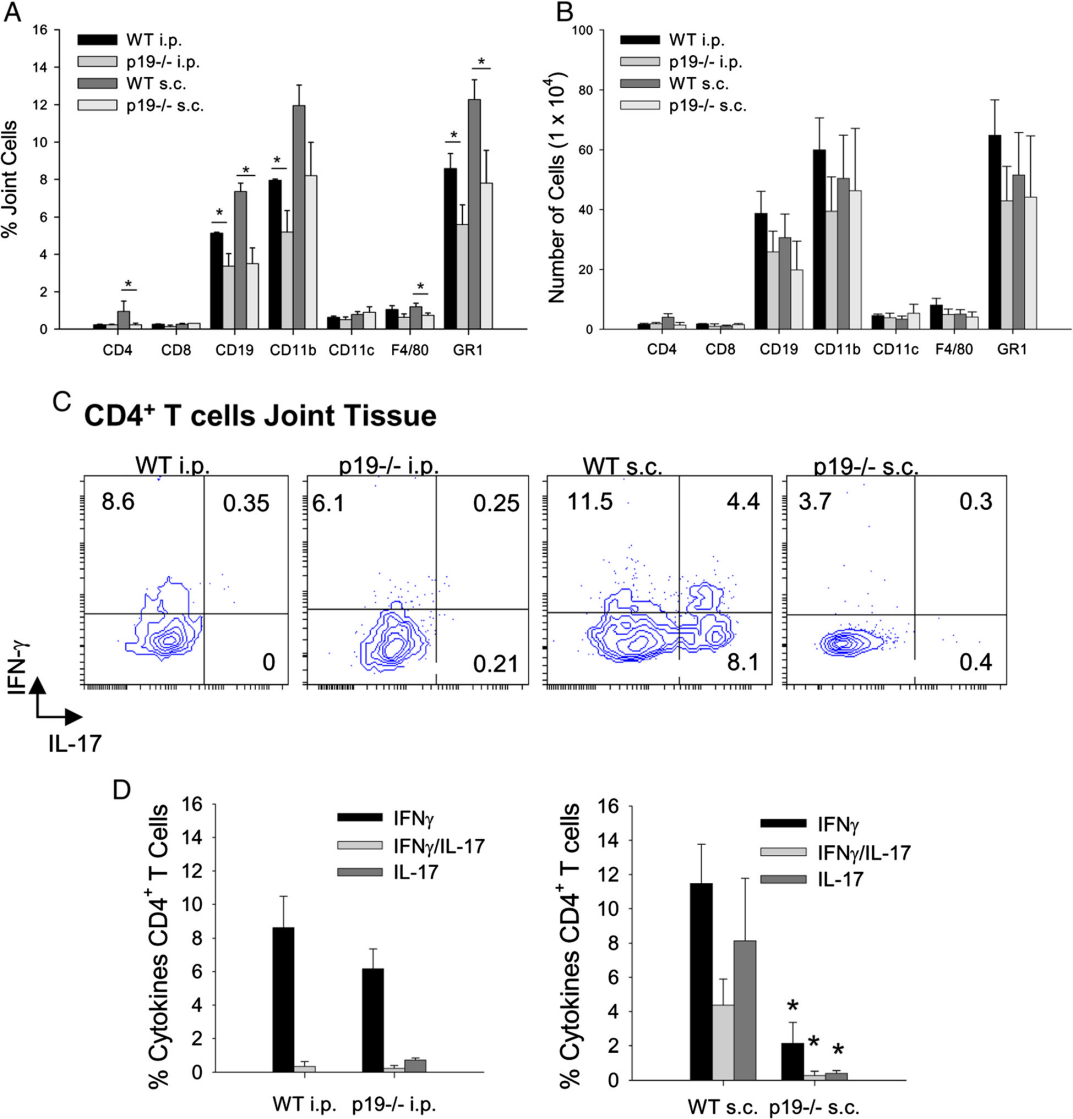
**IFNγ, IL-17 and double-positive IFNγ/IL-17 cytokines in joint tissues are reduced in IL-23p19** ^**−/−**^ **mice after subcutaneous immunization.** Cells were obtained from ankle joints minced, treated with collagenase, and stained with specific antibodies. **(A)** Joint cells (%) and **(B)** number of cells identified by flow cytometry. Data represent mean ± standard error of the mean (SEM, *n* =4 mice). *P <0.05. **(C)** Representative flow cytometry plots of gated CD4^+^ T cells expressing IFNγ and IL-17 from joint tissues of wild type (WT) and IL-23p19^−/−^ mice immunized either by the intraperitoneal (i.p.; left two panels) or subcutaneous (s.c.; right two panels) route with rG1 in adjuvant. For intracellular cytokine staining, cells were stimulated with Phorbol 12-myristate 13-acetate and ionomycin for 4 hours. Cells were surface stained for CD4, permeabilized and stained for IFNγ and IL-17 with specific antibodies. **(D)** Bar graphs showing mean ± SEM of percentage of gated CD4^+^ T cells in WT and IL-23p19^−/−^ mice immunized by the i.p. (left panel) and s.c. (right panel) routes expressing IFNγ and IL-17. Data presented as mean ± SEM (*n* =4) from two independent experiments. *P <0.05. IFN, interferon; IL, interleukin.

We next assessed the composition of the cells infiltrating the joint. Joint tissues were recovered from immunized mice, minced and digested with collagenase. The percentage and number of cells were assessed (Figure 2A,B). Neutrophils (GR1^+^CD11b^+^) were the predominant cell population in joint tissues. The percentage of neutrophils was reduced in the p19^−/−^ mice immunized by either the i.p. or s.c. route. There was also a reduction in the percentage of CD19^+^ B cells in p19^−/−^ mice. CD4^+^ T cells were increased in WT s.c. immunized mice in comparison with WT i.p. immunized mice. In s.c. immunized p19^−/−^ mice, the CD4^+^ T cells were reduced. Although there was a trend in the reduction in the numbers of some populations of cells in the joint between WT and p19^−/−^ mice because of the variability between mice, the data did not reach significance. The reduction in CD4^+^ T cells corresponded to the reduction in arthritis severity in p19^−/−^ mice sensitized to antigen by the s.c. route. These results demonstrate that IL-23 is important in arthritis induced by the s.c. route of immunization but plays a less significant role in arthritis induced by the i.p. route.

A population of IFNγ/IL-17 double-positive cells exists in the synovial fluid and synovial tissue of some RA patients [42-44]. To determine whether a similar population was present in joints of arthritic mice, we assessed intracellular cytokine production by the CD4^+^ T cells in joint tissues (Figure 2C,D). Joint tissues were obtained from immunized mice at a time point when the WT mice were arthritic. Joints were homogenized and treated with collagenase to release cells from the tissue. Gated CD4^+^ T cells (Additional file 1) were stained for intracellular IFNγ and IL-17. Interestingly, in WT mice we found a population of IFNγ/IL-17 double-positive cells in joint tissues of s.c. immunized mice that was not present in the i.p. immunized mice. Furthermore, in the p19^−/−^ mice both the single-positive IFNγ and IL-17 CD4^+^ T cells and the double-positive IFNγ/IL-17 CD4^+^ T cells were reduced (Figure 2C,D). These data demonstrate that IL-23 drives both IL-17-expressing and IFNγ-expressing T cells in arthritic joint tissues of s.c. immunized mice.

### IL-17 and IFNγ are reduced in spleen and inguinal lymph node in IL-23p19^−/−^ mice after subcutaneous immunization

**Figure 3:**
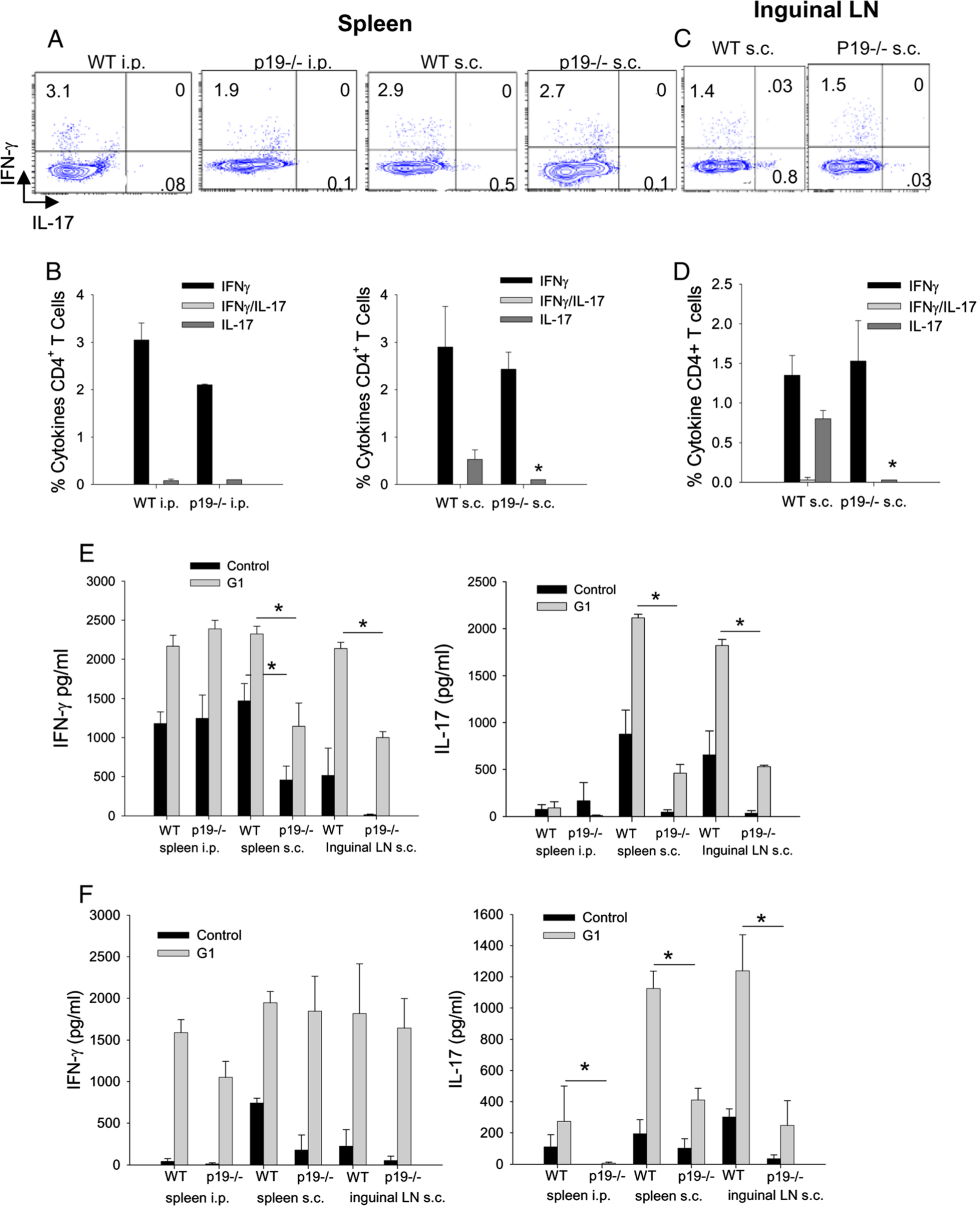
**CD4** ^**+**^ **T cell IL-17 and IFNγ production in spleen and lymph node is inhibited in subcutaneous immunized IL-23p19** ^**−/−**^ **mice.** Spleen and lymph node (LN) were harvested approximately 70 days after rG1/dimethyldioctadecyl ammonium bromide immunization. **(A)** Representative flow cytometry images of CD4^+^ T cells from the spleen of BALB/c wild type (WT) and p19^−/−^ intraperitoneal (i.p.; left two panels) and subcutaneous (s.c.; right panels) immunized mice with rG1in adjuvant. **(B)** Percentage of CD4^+^ T cells expressing cytokines in spleen after i.p. (left panel) and s.c. (right panels) immunization. **(C)** Representative flow cytometry images of CD4^+^ T cells from inguinal LN of BALB/c WT and p19^−/−^ s.c. immunized mice. **(D)** Percentage of CD4^+^ T cells expressing cytokines in inguinal LN after s.c. immunization. **(E)** Concentration (pg/ml) of IFNγ (left panel) and IL-17 (right panel) from CD4^+^ T cells from WT and p19^−/−^ mice after either i.p. or s.c. immunization and cultured in the presence of rG1and antigen-presenting cells. Supernatants were harvested on day 4 and assayed for cytokines by enzyme-linked immunosorbent assay. Data represent mean ± standard error of the mean (SEM, *n* =5 mice) from two independent experiments. **(F)** IFNγ (left panel) and IL-17 (right panel) concentration (pg/ml) from purified CD4^+^ T cells restimulated in the presence of rG1 and antigen-presenting cells for 4 days. BALB/c WT and p19^−/−^ mice were immunized with rG1 either by i.p. or s.c. route and spleen and inguinal LN were harvested on day 9. Data are the mean ± SEM (*n* =4 mice) and representative of three independent experiments. *P <0.05. IFN, interferon; IL, interleukin.

We next determined whether IL-23 was required for generating IFNγ/IL-17 double-positive CD4^+^ T cells in peripheral lymphoid tissues. Spleen and inguinal LN were harvested from WT and p19^−/−^ mice approximately 70 days after the initial immunization with rG1/adjuvant. CD4^+^ T cells were examined for IFNγ and IL-17 expression by intracellular staining. We observed only single-positive IFNγ and IL-17 CD4^+^ T cells from the spleen (Figure 3A,B) and inguinal LN (Figure 3C,D). There were no IFNγ/IL-17 double-positive CD4^+^ T cells after either i.p. or s.c. immunization as was observed in the joint tissue from s.c. immunized mice. In i.p. or s.c. immunized mice, the percentage of CD4^+^ T cells expressing IFNγ was not reduced in p19^−/−^ mice in either spleen or inguinal LN (Figure 3B,D). There was a small percentage of CD4^+^ T cells expressing IL-17 that was significantly reduced in p19^−/−^ in spleen and inguinal LN after s.c. immunization (Figure 3B,D). When we assessed secreted cytokines from spleen and inguinal LN CD4^+^ T cells activated in the presence of rG1 and antigen-presenting cells, the IFNγ response was not inhibited in i.p immunized p19^−/−^ mice; however, it was significantly inhibited in both spleen and inguinal LN after s.c. immunization (Figure 3E). As previously reported there is almost no IL-17 produced after i.p. immunization whereas substantial IL-17 production was observed after s.c. immunization in both spleen and inguinal LN, which was significantly inhibited in p19^−/−^ mice (Figure 3E). These data confirm the previous work showing that IL-23 is necessary for the maintenance of the IL-17 response. In addition, the reduction in IL-17 correlated with the suppression in arthritis after immunization of p19^−/−^ mice by the s.c. route. Although we did not find a significant reduction in intracellular IFNγ in p19^−/−^ mice, IFNγ supernatants from CD4^+^ T cells activated *in vitro* were reduced, suggesting that the amount of IFNγ produced per IFNγ-producing T cell was inhibited. Interestingly, we found that the IFNγ response was also inhibited in p19^−/−^ mice after s.c. immunization, unlike after i.p. immunization, suggesting that IL-23 regulates the amount of IFNγ as well as IL-17.

To determine whether chronic activation *in vivo* was required for development of the IL-23-dependent IFNγ response, we immunized mice by either the i.p. or s.c. route and harvested spleen and inguinal LN early after activation on day 9. CD4^+^ T cells from WT and p19^−/−^ mice after i.p. and s.c. immunization were activated *in vitro* with rG1 and supernatants harvested on day 4. There was no significant difference in the IFNγ produced by WT or p19^−/−^ CD4^+^ T cells whereas, as expected, the IL-17 response was significantly inhibited in p19^−/−^ mice (Figure 3F). These data demonstrate that chronic activation *in vivo* is necessary for the dependence of IFNγ on IL-23 after s.c. immunization.

### CD11c^+^ dendritic cells express IL-23 that drives T-cell production of IL-17

**Figure 4:**
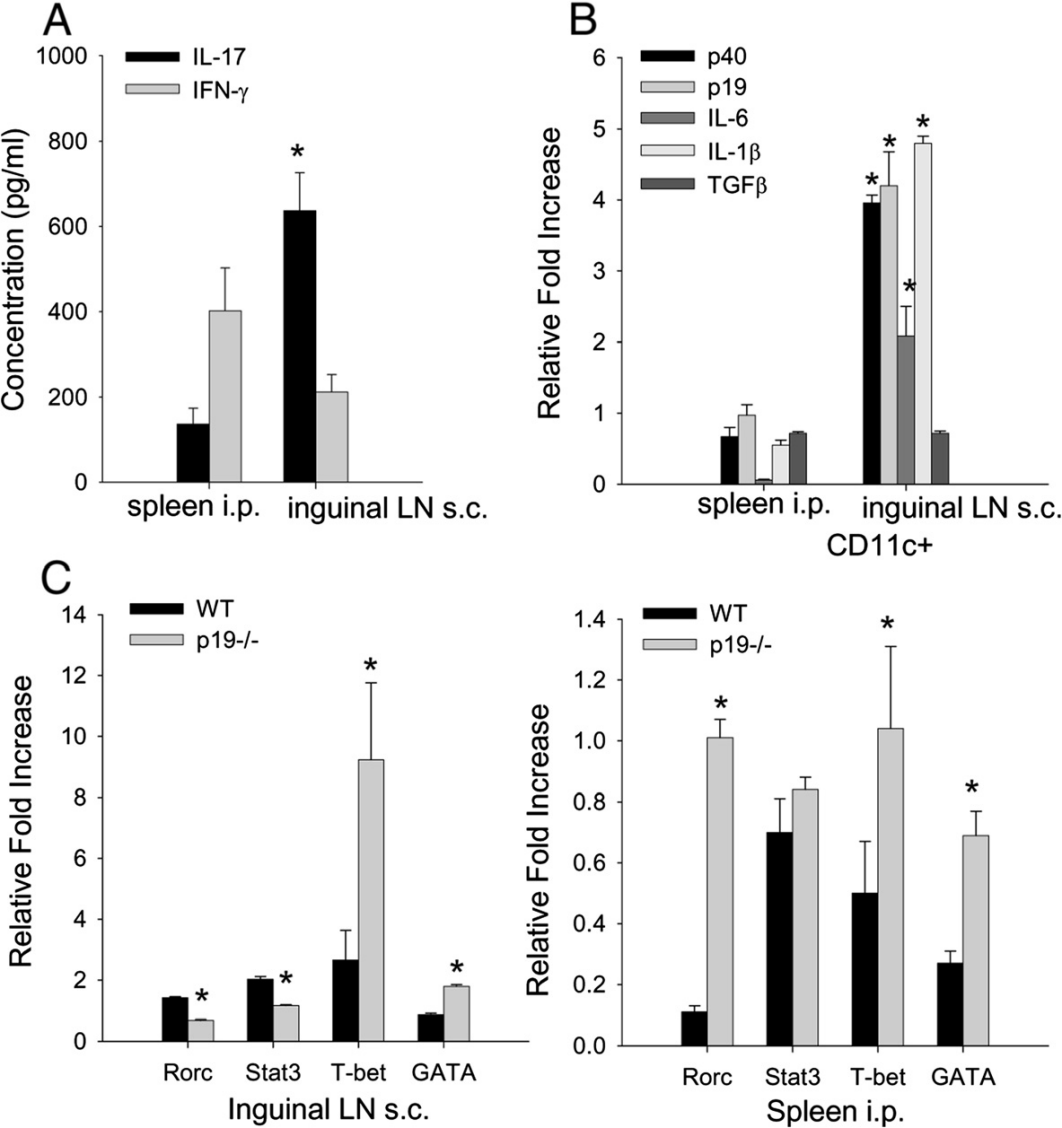
**Cytokines and transcription factors expressed by inguinal lymph node CD11c** ^**+**^ **dendritic cells activate Th17 cells. (A)** BALB/c wild type (WT) mice were immunized by the intraperitoneal (i.p.) and subcutaneous (s.c.) routes and at 24 hours spleen and inguinal lymph node (LN) were harvested and CD11c^+^ dendritic cells (DCs) sorted and cultured with TCR-Tg 5/4E8 CD4^+^ T cells. Supernatants were assayed for IFNγ and IL-17 by enzyme-linked immunosorbent assay. **(B)** RNA isolated from CD11^+^ DCs from i.p. spleen and s.c. inguinal LN at 6 hours (p19) and 12 hours (p40, IL-6, IL-1β, and TGFβ) after immunization was used to assess RNA transcripts. Bar graph represents the relative fold increase in transcript expression. CD11c^+^ DCs from nonimmune spleen and inguinal LN were used to calculate the relative fold increase in cytokine RNA expression. **(C)** BALB/c WT and p19^−/−^ were i.p. and s.c. immunized. RNA was isolated from i.p. spleen and s.c. inguinal LN on day 5 and assessed for mRNA transcripts for transcription factors. Bar graph represents relative fold increase in transcription factors from s.c. inguinal LN (left panel) and i.p. spleen (right panel). Nonimmune spleen and inguinal LN were used to calculate the relative fold increase in transcription factor expression. Data are presented as mean ± standard deviation (*n* =3 to 6) *P <0.05. IFN, interferon; IL, interleukin; TGFβ, transforming growth factor beta.

We have previously reported that antigen-presenting cells from the spleen after exposure to antigen by the i.p. route activate T cells to produce IFNγ but very little IL-17. To determine whether CD11c^+^ DCs were involved, we sorted CD11c DCs from the spleen 24 hours after i.p. immunization and from inguinal LN after s.c. sensitization and activated naïve CD4^+^ T cells from TCR-Tg 5/4E8 mice. CD11c^+^ DCs from the spleen of i.p. immunized mice activated T cells to produce significantly less IL-17 than T cells stimulated with CD11c^+^ DCs from inguinal LN of s.c. immunized mice, whereas there was no difference in IFNγ production (Figure 4A).

To determine whether CD11c^+^ DCs expressed different cytokines after i.p. and s.c. sensitization, we examined several cytokines that are important for Th1 and Th17 differentiation. Mice were i.p. and s.c. immunized with rG1 in adjuvant and CD11c^+^ DCs isolated from the spleen (i.p.) and inguinal LN (s.c) at 6-hour and 12-hour time points.

CD11c^+^ DCs isolated from spleen (i.p.) sensitized for 12 hours expressed minimal mRNA transcripts for p40, IL-6, IL-1β, and TGFβ, whereas CD11c^+^ DCs from inguinal LN after s.c. sensitization expressed significant increased mRNA transcripts for p40, IL-1β, IL-6, but not TGFβ (Figure 4B). At the 6-hour time point, p19 mRNA transcript was expressed from s.c. sensitized inguinal LN but not i.p. sensitized spleen (Figure 4B).

We have shown that IL-23p19 is necessary for the expression of IL-17 predominantly after s.c. immunization. We next assess whether transcription factors associated with differentiation of Th1 and Th17 cells were altered in p19^−/−^ mice. WT and p19^−/−^ mice were i.p. and s.c. immunized and the spleen and inguinal LN were isolated on day 5. Transcription factor transcripts were assessed directly without restimulation *in vitro*. In the inguinal LN, Rorc and Stat3 transcription factors were inhibited in p19^−/−^ mice in comparison with WT. Interestingly, T-bet and GATA3 transcripts were increased in p19^−/−^ mice compared with WT mice (Figure 4C). These data demonstrate that the reduction in IL-17 in the p19^−/−^ s.c. sensitized mice is through reduction in Rorc and Stat3. It has been shown that IL-23 can inhibit IFNγ [45]. Our data suggest this may occur through T-bet. IL-23 may also inhibit IL-4 as GATA3 is upregulated in p19^−/−^ mice. In the spleen, after i.p. immunization, transcripts Rorc, T-bet, Stat3, and GATA3 were reduced in WT mice relative to naïve mice normalized to one. In p19^−/−^ mice, Rorc, T-bet, and GATA3 but not Stat3 were significantly increased in comparison with WT mice, suggesting that IL23p19 was involved in the reduction in these transcription factors (Figure 4C).

## Discussion

This study was designed to determine whether IL-23 has an effect on the development of arthritis independent of IL-17. PGIA is critically dependent on T cells because T cells are required for the successful transfer of disease in T-cell-deficient mice. [46]. PGIA is considered a Th1-mediated disease based on the evidence that components of the Th1 pathway, IFNγ, p40, p35, T-bet, Stat4 and IL-27 receptor are necessary for the development of PGIA [33-37]. We discovered that the Th1 phenotype in PGIA is based on the induction of disease by immunization with PG by the i.p. route [38]. It was found that i.p. immunization induces IFNγ but very little IL-17, and few CD4^+^ T cells produce IL-17. Altering the route of immunization to the s.c. route that is used in EAE, EAU, and CIA converts PGIA to IL-17-dependent arthritis where both IFNγ-producing and IL-17-producing CD4^+^ T cells are activated. In the present study, we provide evidence that, similar to CIA, severe arthritis induced by the s.c. route of immunization was dependent on IL-23. In the p19^−/−^ and p40^−/−^ mice immunized with rG1 by the s.c. route, there was very mild disease in a few mice. In contrast to p40^−/−^ mice, p19^−/−^ mice immunized by the i.p. route exhibited joint swelling and erythema although it was less severe than in WT mice. We reported previously that p35^−/−^ mice are resistant to developing arthritis by the i.p. route [36]. We would anticipate that p35^−/−^ mice are susceptible to developing arthritis induced by s.c. immunization similar to IFNγ^−/−^ mice [38]. Our data suggest that IL-23 has some effect on arthritis after i.p. immunization. The histological picture of disease was similar to WT although there were fewer infiltrating cells in the joint. We previously were unable to identify a role for IL-17 after i.p. immunization because IL-17-deficient mice exhibited a similar degree of arthritis as WT mice [36]. Thus, IL-23 may have other affects on the development of arthritis such as the induction of IL-1β and IL-6 [47]. IL-23 also mediates activities independent of IL-17. IL-23 mediates osteoclastogenesis by induction of receptor activator of nuclear factor-κB and cathepsion K expression [31]. Also, systemic induction of IL-23 induces entheseal inflammation that is dependent on IL-22 [32]. IL-22 is produced by Th17 in an IL-23-dependent manner or by a T-cell lineage cell, Th22 [48,49]. However, in a model of infection with *Mycobacterium tuberculosis*, the IL-22 cells are dependent on IL-23 and the majority of the IL-22-producing T cells also express IFNγ [50]. The reduction in arthritis in p19-deficent mice induced by the i.p. route may thus be due to IL-22 or other inflammatory cytokines induced by IL-23.

We show here that the CD4^+^ T cells in the joints from s.c. sensitized mice produce single-positive IL-17 and IFNγ and double-positive IL-17/IFNγ T cells, and expression of both IFNγ and IL-17 was dependent on IL-23. Systemically, in chronically activated s.c. sensitized mice the IL-17/IFNγ T cells were not present in the inguinal LN; however, both the IL-17 and IFNγ single-positive cells were dependent on expression of IL-23. The dependence of IFNγ on IL-23 was due to chronic activation because acute activation (day 9 sensitization) was independent of IL-23. Recent data indicate that Th17 cells activated in the presence of IL-23 and low or absent TGFβ are unable to maintain IL-17 expression and transition to IFNγ-expressing T cells [51]. However, studies that linked IL-23 to induction of IL-17 expression by memory CD4^+^ T cells also demonstrate induction of IFNγ expression. T cells expressing IL-17/IFNγ are found in the context of IL-17-dependent autoimmune diseases [17,51] and IL-23 is necessary to sustain the IL-17/IFNγ phenotype [51,52]. In RA patients expressing IL-17 and IFNγ, a population of double-positive IFNγ/IL-17 producers is present similar to our observation in mice immunized by the s.c. route [42,43,53-55]. It is presently unclear whether IL-17/IFNγ T cells present in some RA patients are dependent on IL-23. In RA patients, elevated levels of p19 and p40 subunits are found in synovial fluid and sera [56,57]. However, when the heterodimeric IL-23 (p19/p40) is measured, very low levels are found despite high levels of the p19 subunit [58]. These findings suggest that the presence of the p19 subunit does not necessarily indicate the presence of IL-23. An association of IL-23 levels in plasma with disease severity is shown in early RA but not in chronic RA [59,60]. In RA patients, some but not other local parameters of joint inflammation are found associated with IL-23 [61]. The importance of IL-23 in IL-17 expression is suggested by their co-expression in some patients and the increase in IL-23p19 in patients that are IL-17-positive [62,63]. Studies in RA showed that IL-17-producing T cells are significantly increased in RA patients in comparison with OA or normal controls [44,62,64-66]. Current evidence suggests that IL-17 amplifies disease in the subset of patients in which it is present. In several studies there is a significant correlation between IL-17 levels and progression to arthritis or a more aggressive form of disease or C-reactive protein levels [67-70]. It is important to note that not all patients express IL-17, however, indicating it is not absolutely required for inflammation in RA [53,60,66,67,71]. Several IL-17A inhibitors are being tested in clinical trials. Treatment with monoclonal antibodies to IL-17A (secukinumab and ixekizumab) show favorable responses in some but not all RA patients [72]. RA patients may thus be divided into subtypes based on expression of the IL-23/IL-17 pathway.

We reported previously and confirm here that T cells from i.p. sensitized mice produce very little IL-17 and abundant IFNγ whereas T cells from s.c. sensitized mice produce both IL-17 and IFNγ in response to antigen restimulation *in vitro*. The increase in IL-17 production was not the result of a more potent immune response to s.c. sensitization, as the inguinal LN and splenocytes of s.c. and i.p. immunized mice secreted similar amounts of IFNγ in response to antigen. Since IL-17 can be inhibited by IFNγ, this finding suggests that the selective induction of IL17 by s.c. immunization is not due to decreased secretion of IFNγ. Thus, it appears that s.c. sensitization may evoke a response in the skin or draining LN that is in some way different from i.p. sensitization in the peritoneal cavity and spleen. One way this could occur is suggested by a recent report demonstrating that the environment of the peritoneal cavity may recruit Th1 cells, because the peritoneal cavity appears to be dominated by IFNγ^+^CXCR3^+^ T cells in naïve mice [73]. Since it is known that IFNγ can suppress IL-23 [74], the programming of the peritoneal cavity to produce IFNγ may block the differentiation of Th17 cells. We found that IFNγ contributes to the reduced IL-17 responses because in IFNγ^−/−^ mice IL-17 is increased after i.p. sensitization and PGIA is inhibited more effectively in IFNγ/IL-17^−/−^ mice than in IFNγ^−/−^mice [37]. Further studies will be needed to address this issue.

CD11c^+^ DCs from s.c. sensitized inguinal LN induced a T-cell IL-17 response whereas the IL-17 response was significantly less if the CD11c^+^ DCs were from the spleen of i.p. sensitized mice. In addition, CD11c^+^ DCs from inguinal LN strongly upregulated cytokine transcripts for p19, p40, IL-6, and IL-1β in comparison with CD11c^+^ DCs from splenocytes of i.p. immunized mice. These results suggest that the increase in production of cytokines by CD11c^+^ DCs that drive Th17 differentiation are preferentially produced by s.c. sensitization. The peripheral LN contains several migratory DC populations that are absent in the spleen. We reported previously that a population of CD11c^+^CD11b^+^CD103^+^ DCs takes up labeled G1 after s.c. sensitization [38]. These DCs may have the capacity to induce Th17 differentiation as similar DCs that induce IL-17 are found in the intestines [75]. In contrast, we found that the spleen contains a population of CD11c^+^CD11b^+^ CD103^−^CD205^−^CD8^−^ cells that takes up labeled G1 after i.p. sensitization. Similar DCs are a source of IL-12 in *Listeria*-infected mice [76]. In addition to different DCs that are present in the spleen and inguinal LN, the tissue microenvironment is thought to have a major impact on the nature of immune responses induced at difference tissue sites. Studies examining colon, lung, and spleen CD11c^+^ DCs found that all tissues have typical antigen-presenting function; however, they exhibited distinct cytokine production profiles, modulation of T-cell responses, regulatory T-cell induction, and different toll-like receptor expression patterns [77,78].

In assessing the transcription factors associated with IFNγ and IL-17 response in p19^−/−^ mice we found that, as expected, Rorc and Stat3 expression was inhibited in the s.c. sensitized inguinal LN of p19^−/−^ mice. However, in the spleen after i.p. sensitization, Rorc, Stat3, T-bet, and GATA3 transcripts from WT mice were reduced relative to nonimmunized naïve mice. In p19^−/−^ mice, Rorc, Stat3, and GATA3 were enhanced suggesting that IL23p19 may play a role in inhibiting these transcripts in WT mice after i.p. exposure to antigen. It is presently unclear how the loss of p19^−/−^ leads to an increase in Rorc. T-bet is increased in both inguinal s.c. LN and i.p. spleen in p19^−/−^ mice. IL-23 is known to inhibit IFNγ, and thus T-bet may be involved in this process [45]. Interestingly, GATA3 transcripts were also increased in p19^−/−^ mice. Although not reported previously, IL-23 may also inhibit IL-4 through suppressing GATA3.

Our observation that PGIA induced by immunization at different anatomical sites is associated with distinct immunological mechanisms (Th1 or Th17 dependent) is both novel and has the potential to alter the paradigm by which RA is evaluated and treated, as directed therapies might be more rationally selected in individual patients based upon the mechanism underlying their disease. Our model is the only one known that shows arthritis is induced differently when immunizing with the same antigen via different routes. Clinically, RA appears to be mediated by particular genetic susceptibility combined with environmental interactions (infectious agents or environmental stimuli). These triggers could occur in different individuals at different anatomical sites in the body depending on the site; that is, lung (smoking), skin (infection) or peritoneal cavity (gastrointestinal leakage). For example, RA often involves multiple body systems other than the joints. Interstitial lung disease in some cases precedes joint disease, suggesting the lung as a possible site of initiation of the immune dysregulation that leads to clinical RA [79]. Similarly, there is evidence that periodontal disease and inflammatory bowel disease-associated inflammatory polyarthropathy may be initiated at these distinct anatomical sites [80,81]. The initiation of PGIA by different routes may mimic these different modes of induction of RA, and is an innovative and unique tool to study immunological processes associated with arthritis. This model permits a systematic analysis of whether distinct mechanisms are associated with different responsiveness to therapies, and may identify a unique signature associated with these pathways.

## Conclusions

We would suggest that differential activation of Th1 versus Th17 phenotype may in part be responsible for different subtypes of RA patients. Since T cells play an important role in RA and infection or injury is associated with disease flares in RA, it is possible that different compartments or routes of antigen exposure could affect the dominance of Th1 versus a Th17 response. The different animal models of RA may represent unique subtypes and may help us understand disparate clinical responses to better individualize therapeutic choices.

## Additional file

Additional file 1:
**shows the gating of CD4** ^**+**^ **T cells for assessment of IFNγ and IL-17.** Joint tissues were minced and treated with collagenase (0.25 mg/ml), and joint cell populations were examined for surface markers using antibodies. For intracellular cytokine staining, cells were stimulated with Phorbol 12-myristate 13-acetate (25 ng/ml) and ionomycin (500 ng/ml) (Sigma-Aldrich) and treated with GolgiPlug (BD Pharmingen) for 4 hours. After cell surface staining with ant-CD3e-PE-Cy7 anti-CD4-APC-Cy7, cells were permeabilized using the Cytofix/Cytoperm Plus kit (BD Pharmingen) and stained with anti-IFN-γ-APC and anti-IL-17A-FITC.

## Abbreviations

CIA: collagen-induced arthritis
DC: dendritic cell
EAE: experimental autoimmune encephalomyelitis
EAU: experimental autoimmune uveitis
IFNγ: interferon gamma
IL: interleukin
i.p.: intraperitoneal
LN: lymph node
PG: proteoglycan
PGIA: proteoglycan-induced arthritis
RA: rheumatoid arthritis
rG1: recombinant human aggregan G1 domain
s.c.: subcutaneous
TGFβ: transforming growth factor beta
Th: T-helper
WT: wild type
