# Supplementary material for: Tissue specific CD4+ T cell priming determines the requirement for interleukin-23 in experimental arthritis
Source: Arthritis Res Ther. 2014 Sep 25;16(5):440. doi: 10.1186/s13075-014-0440-1 (PMC4203961; doi:10.1186/s13075-014-0440-1)
Supplement: Additional file 1: — shows the gating of CD4 + T cells for assessment of IFNγ and IL-17. Joint tissues were minced and treated with collagenase (0.25 mg/ml), and joint cell populations were examined for surface markers using antibodies. For intracellular cytokine staining, cells were stimulated with Phorbol 12-myristate 13-acetate (25 ng/ml) and ionomycin (500 ng/ml) (Sigma-Aldrich) and treated with GolgiPlug (BD Pharmingen) for 4 hours. After cell surface staining with ant-CD3e-PE-Cy7 anti-CD4-APC-Cy7, cells were permeabilized using the Cytofix/Cytoperm Plus kit (BD Pharmingen) and stained with anti-IFN-γ-APC and anti-IL-17A-FITC. [file 13075_2014_440_MOESM1_ESM.pdf]

Additional file 1

Gating of CD4+ T cells for assessment of IFN-γ and IL-17

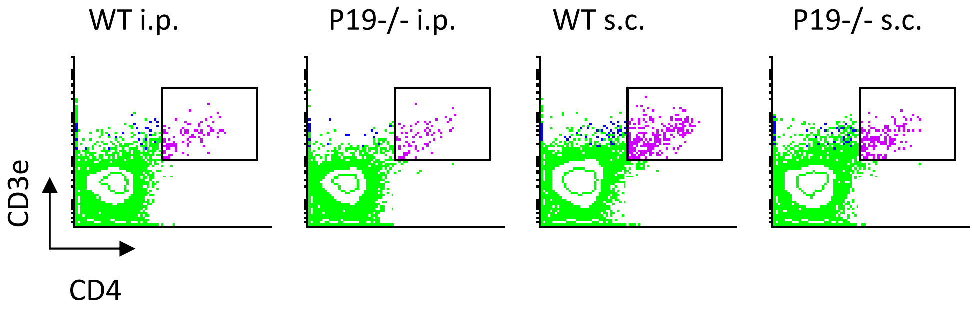

Joint tissues were minced, treated with collagenase (0.25mg/ml) and joint cell populations examined for surface markers using antibodies. For intracellular cytokine staining, cells were stimulated with PMA (25 ng/ml) and ionomycin (500ng/ml) (Sigma-Aldrich) and treated with GolgiPlug (BD Pharmingen) for 4 h. After cell surface staining with ant-CD3e-PE-Cy7 anti-CD4-APC-Cy7, cells were permeabilized using the Cytofix/Cytoperm Plus kit (BD Pharmingen) and stained with anti-IFN-γ-APC and anti-IL-17A-FITC.
